# Alternative Approaches to Adenotonsillectomy and Continuous Positive Airway Pressure (CPAP) for the Management of Pediatric Obstructive Sleep Apnea (OSA): A Review

**DOI:** 10.1155/2020/7987208

**Published:** 2020-07-04

**Authors:** Mandeep Rana, Joshua August, Jessica Levi, Goli Parsi, Melih Motro, William DeBassio

**Affiliations:** ^1^Department of Pediatrics, Division of Pediatric Neurology and Sleep Medicine, Boston University School of Medicine, Boston Medical Center, Boston, MA 02118, USA; ^2^Department of Otolaryngology, Boston University School of Medicine, Boston Medical Center, Boston, MA 02118, USA; ^3^Boston University Dentistry, Boston University Henry M. Goldman School of Dental Medicine, Boston Medical Center, Boston, MA 02118, USA

## Abstract

Continuous positive airway pressure (CPAP) is considered first-line treatment in the management of pediatric patients without a surgically correctible cause of obstruction who have confirmed moderate-to-severe obstructive sleep apnea (OSA). The evidence supports its reduction on patient morbidity and positive influence on neurobehavioral outcome. Unfortunately, in clinical practice, many patients either refuse CPAP or cannot tolerate it. An update on alternative approaches to CPAP for the management of OSA is discussed in this review, supported by the findings of systematic reviews and recent clinical studies. Alternative approaches to CPAP and adenotonsillectomy for the management of OSA include weight management, positional therapy, pharmacotherapy, high-flow nasal cannula, and the use of orthodontic procedures, such as rapid maxillary expansion and mandibular advancement devices. Surgical procedures for the management of OSA include tongue-base reduction surgery, uvulopalatopharyngoplasty, lingual tonsillectomy, supraglottoplasty, tracheostomy, and hypoglossal nerve stimulation. It is expected that this review will provide an update on the evidence available regarding alternative treatment approaches to CPAP for clinicians who manage patients with pediatric OSA in daily clinical practice.

## 1. Introduction

Obstructive sleep apnea (OSA) is characterized by intermittent partial or complete upper airway closure during sleep, associated with increased respiratory effort, sleep fragmentation, and/or gas exchange abnormalities [1]. The decision to treat children with OSA is made based on the age of the child, the severity of symptoms, the clinical findings, the presence of comorbidities, and the PSG findings [2]. Adenotonsillectomy (AT) is recommended for otherwise healthy children who have OSA and adenotonsillar hypertrophy [3]. However, the prevalence of residual OSA following AT in children has been reported to be as high as 25% and 40% [4, 5]. In symptomatic or moderate-to-severe OSA in the absence of enlarged tonsils, the first-line treatment option is positive airway pressure treatment either continuous or bilevel [6]. Continuous positive airway pressure (CPAP) applies a constant level of pressure in the airway throughout the respiratory cycle, whereas bilevel positive airway pressure uses a higher level of positive airway pressure during inspiration than expiration [6, 7]. For each child, fitting of the CPAP equipment and adjustment of the pressure settings, should be performed in a sleep laboratory by healthcare professionals with expertise in the management of pediatric patients. Continued monitoring of CPAP equipment with periodic PSG evaluation is required in the event the child's symptoms change or if their body mass index (BMI) increases [7]. Several observational clinical studies have shown that faithful usage of CPAP can improve symptoms evident on improved PSG findings in up to 85% of children diagnosed with OSA [7, 8]. The most common side effects include dry mouth, increased number of awakenings, blocked nose, and mask leaks arising from a poorly fitting mask [9]. Long-term effects from pressure to the midface altering normal facial growth have been reported [9]. The main concern regarding CPAP for the treatment of OSA in children is the high prevalence of noncompliance and refusal to use the device (25% to 50%) [10, 11].

The aim of this review is to examine non-CPAP options for the management of OSA. These include weight loss, positional therapy, pharmacotherapy, and the use of orthodontic procedures, including rapid maxillary expansion (RME) and mandibular advancement devices (MADs). Surgical procedures for the management of OSA include tongue-base reduction surgery, uvulopalatopharyngoplasty (UPPP), lingual tonsillectomy, and tracheostomy. The use of high-flow nasal cannula (HFNC) therapy and hypoglossal nerve stimulation therapy are discussed. Interventions for children with OSA are determined by a variety of parameters on a case-by-case basis, including age, severity of symptoms, presence of complex abnormalities or conditions (such as obesity, craniofacial deformities, malocclusion, neuromuscular disorders, Down's syndrome, and Prader-Willi syndrome) and treatment thereof, degree of residual disease following treatment, and accepted practices in patients' respective countries [12, 13]. This review is not intended to provide recommendations for clinical practice but to examine the evidence for possible treatments in the pediatric patient population, and in particular, to provide an update on the evidence to support alternative treatment approaches to adenotonsillectomy and CPAP for clinicians who manage patients with OSA.

## 2. Weight Management

As OSA is caused by pharyngeal collapse or dysfunction, the deposition of pharyngeal fat in obese children may explain why obesity is a risk factor. Weight loss efforts are considered adjunctive therapy in the management of pediatric OSA, but only in children who are overweight or obese [14–16]. The 2011 recommendations of the interdisciplinary European Respiratory Society Task Force concluded that weight reduction was associated with improvements in breathing pattern, improvement of quality of sleep, and reduced daytime sleepiness. Many children with OSA, however, are of normal weight or are underweight, and so careful monitoring of height and weight are required before weight loss is recommended [14]. The view that weight loss is beneficial to children with OSA was derived from evidence based on clinical studies in overweight adults [17].

In 2009, Verhulst et al. assessed the effect of weight loss on SDB in 61 obese teenagers with a mean age of 14.8 ± 2.3 years and an AHI ≥ 2. Weight loss was successful in reducing symptoms. There was a positive association between the severity of OSA at the start of the treatment, the amount of weight loss achieved, and a concomitant reduction of symptoms [18]. This study is limited by the small number of subjects. A laboratory sleep study had not been obtained; thus, arousal-based events may have been missed. In addition, weight loss in study subjects was generated by a residential treatment program and was not comparable with programs in outpatient clinics.

Recently, Xanthopoulos and colleagues reviewed the three main forms of obesity therapy, which include lifestyle modification, the use of pharmacologic agents, and bariatric surgery. The conclusions were that individuals have a highly variable response to weight loss interventions and that maintaining weight loss remains a challenge. Particularly in children, weight loss strategies must be individualized [18].

In adults with OSA, obesity, and diabetes, the Sleep AHEAD Study is the most comprehensive randomized controlled clinical study to date to compare diet combined with intensive lifestyle intervention (ILI) and diabetes education. A 10% reduction in initial weight was associated with a 20% improvement in AHI at one year [19]. However, there have been no similar controlled studies on lifestyle intervention combined with weight loss in the pediatric population with OSA.

Bariatric surgery for the treatment of obesity in children should be considered only in cases of severe OSA [14]. A recent controlled study on the clinical course of OSA in adolescents and young adults after bariatric surgery, including vertical sleeve gastrectomy or gastric bypass, used PSG before, at 3 weeks and 5 weeks after surgery. Before surgery, the mean age of the study participants was 17.8 years (range, 15.4–20.7 years), the mean BMI was 55.2 kg/m^2^ (range, 41.3–61.6 kg/m^2^), and the mean AHI was 15.8 events/hour (7.1–23.8 events/hour). Following bariatric surgery, the AHI declined from baseline by 9.2 events/hour (95% CI, 3.8–14.5) by 3 weeks (*P* = 0.002) and 9.1 events/hour (95% CI, 3.8–14.5) at 5 weeks (*P* = 0.002), with no significant change in AHI from three to five weeks, and leptin levels significantly decreased by 3 weeks postoperatively. The findings from this study indicated that OSA responds early and significantly following bariatric surgery and that factors independent of weight might be partly responsible for early postoperative improvement in OSA [20].

Weight loss from different modalities may be of benefit on OSA. There is a clear need for well-controlled clinical studies to evaluate the short- and long-term efficacy of different approaches to the management, the degree of weight loss, and their effects on OSA in children [14, 18]. How the effects of weight loss compared with CPAP in the treatment of OSA must also be further explored.

## 3. Pharmacotherapy

In 2006, two published reviews of medical therapy for pediatric OSA, including a review from the AASM, concluded that there were no effective medical treatments at that time. These authors also reported a lack of knowledge of neurochemical mechanisms associated with OSA, and that well-controlled, adequately powered clinical studies were required that include the effects of pharmacotherapy on AHI and all health-related outcomes when compared with CPAP [21, 22]. Since 2006, there have been several developments in pharmacotherapy for OSA in children, resulting in a range of treatment and drug delivery approaches [23].

Several studies have supported the beneficial effects of intranasal corticosteroid therapy in children with mild OSA [21, 23]. However, the use of systemic corticosteroids has not been shown to be effective in children with OSA, as demonstrated by the findings from an open-label clinical trial that included 5 days of treatment with oral prednisone, which did not improve PSG parameters or reduce the severity of symptoms of OSA [24].

The findings of one of the first randomized trials addressing children with OSA who were treated with intranasal corticosteroid therapy included 25 children treated intranasally with corticosteroids or with placebo for 6 weeks [25]. Treatment with intranasal corticosteroids for 6 weeks resulted in a modest improvement in the symptoms of OSA and reduced the AHI reduction 11 to 6 events per hour [25]. These findings were supported by those from a double-blind, randomized trial that included 48 children with mild OSA treated with intranasal budesonide [26]. The results showed that after 1 week of treatment, the PSG normalized in 50% of the children included in the budesonide treatment arm. This study also found that the beneficial effects of intranasal budesonide treatment were present up to 8 weeks after the end of the study [26]. A systematic review that included seven controlled studies and 493 children showed significant efficacy of intranasal corticosteroid treatment in improving the symptoms of nasal obstruction symptoms, although PSG measurements were not performed [27].

Combined pharmacotherapy may be required in children with OSA [14, 23]. Montelukast is a leukotriene modifier that has been shown to reduce AHI and adenotonsillar size in children with OSA [28, 29]. Children with mild or moderate OSA who have both seasonal allergies and nasal obstruction due to adenoidal hypertrophy may respond to treatment with combined intranasal corticosteroids and leukotriene modifier therapy, as an alternative or adjunctive treatment to AT [30]. The combination of montelukast and intranasal corticosteroids has been shown to be effective in several studies. In a retrospective review of 752 children with mild OSA treated with combined intranasal corticosteroids and montelukast, more than 80% of children experienced subjective beneficial effects, with reduced symptoms of OSA. From this study, 62% of children who underwent follow-up PSG testing showed continued improvement [31]. Combined treatment with intranasal corticosteroids and montelukast was also shown to be effective in children who had previously undergone AT and who had mild residual OSA [32]. Clear guidelines on the duration of treatment required or the long-term benefits of this combined treatment approach remain lacking [21].

Adjunctive therapeutic approaches may be used in addition to pharmacotherapy in children with OSA, including avoidance of allergens and cigarette smoke, and nasal irrigation for children with allergic rhinitis [14].

Although there continue to be developments in pharmacotherapy as an adjunct or alternative to CPAP in children with OSA, the need remains for controlled, large-scale clinical studies on the effects of pharmacotherapy on both subjective and objective measured outcomes, including the AHI and PSG, when compared with CPAP [21, 22].

## 4. Craniofacial Procedures

The upper airway is positioned beneath the cranial base and posterior to the nasomaxillary complex and the mandible. Any deviation from the normal development of these structures will have a direct influence on the size of the upper airway and can predispose towards SDB [33]. Disrupted breathing patterns may in turn have a negative impact on the growth of craniofacial structures contributing to a compromised upper airway [34]. Craniofacial growth is affected by both genetic and functional factors. There have now been several preliminary studies that have shown that orthodontic treatment of abnormalities of the maxilla and mandible may be effective in children with OSA [35]. A summary of these studies is presented in Table 1.

**Figure 1:**
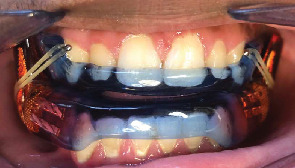
Bilateral interlocking oral appliance in place in a patient.

**Figure 2:**
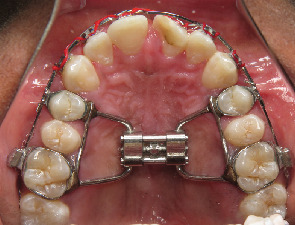
Rapid maxillary expander (4-banded hyrax) in place in a patient.

Procedures that correct craniofacial abnormalities associated with OSA include functional appliances (orthopedic mandibular advancement appliances, Figure 1) and RME (Figure 2) [36–37]. Myofunctional therapy has been reported to be an alternative; however, compliance and long-term outcome in the children remains an issue.

### 4.1. Functional Appliance (Orthopedic Mandibular Advancement Appliances)

Orthopedic mandibular advancement appliances work by protruding the mandible and hence the soft tissue structures attached to it, thus increasing the posterior pharyngeal airway volume and alleviating the tendency of the airway to collapse during sleep. In actively growing children with OSA, the same mechanism of action has been shown to not only improve AHI scores but also to promote growth at the condylar heads, leading to a supplemental increase in overall length of the mandible [38, 39]. In the short term, these appliances have shown improvement in AHI scores. However, there has only been limited published evidence to support these findings. Future studies are needed before it is determined whether this is an effective treatment in pediatric OSA [36].

### 4.2. Rapid Maxillary Expansion

Maxillary expansion, a commonly used orthodontic procedure in growing individuals, is routinely indicated in instances of skeletal and/or dental constriction of the maxilla, unilateral or bilateral crossbite, tooth size/arch-length discrepancy, and dental impactions. In response to maxillary expansion, outer walls of the nasal cavity move laterally in a nonparallel fashion with the greatest expansion occurring in the inferior and anterior areas of the nasal cavity [40]. A recent systematic review [35] of a limited number of articles studying the effects of orthodontic treatment on OSA syndrome in children concluded that individual studies [41]found RME effective in improving snoring and AHI scores. The same findings cannot be extrapolated from the pooled data due to heterogeneity of the subjects and/or intervention. A 12-year follow-up of 23 children with narrow maxilla and no adenotonsillar hypertrophy who were treated with RME showed that the effects on maxillary width were stable and PSG findings stayed normal over the long term [42].

### 4.3. Myofunctional Therapy

This intervention involves teaching patients' specific oropharyngeal exercises to improve labial seal and lip tone, enhance use of nasal breathing as the preferred respiratory route, and promote more favorable positioning of the tongue within the oral cavity [43]. These focused exercises are performed daily and can strengthen the tongue and orofacial muscles, while fostering realignment to the correct intraoral position. However studies are limited by very small number of patients and unclear long-term benefits. Although these exercises are easy to learn and teach, the child's cooperation and adherence is a potential limitation. Recent studies corroborate these concerns and advocate for passive myofunctional therapy via an intraoral appliance rather than active exercise [44].

Although targeted procedures have a role in patients with craniofacial abnormalities, data are limited with regard to their efficacy compared with CPAP in pediatric patients with OSA.

## 5. Surgical Procedures

In children with persistent moderate-to-severe OSA following AT, or those with small tonsils, or unable to tolerate CPAP, a drug-induced sleep endoscopy (DISE) can be performed to assess for other sites of obstruction [45]. In this procedure, a patient is brought to the OR and put to sleep with general anesthesia to mimic night-time sleep. A flexible endoscope is then introduced through the nose to sequentially examine the nasopharynx, pharynx, and larynx. This allows the surgeon to perform site-directed surgery, either at the time of the DISE or at a subsequent operating room visit. Some centers perform a cine MRI instead, during which the child is anesthetized, and an MRI is obtained to assess real-time sites of obstruction simultaneously [5, 46]. As these are relatively new approaches, the ideal anesthetic regimen and grading scale have yet to be determined.

Adjuvant surgical procedures for children with OSA are listed in Table 2. These include UPPP, lingual tonsillectomy, and tongue-base reduction. Tracheotomy is usually undertaken in the presence of severe OSA that has failed to respond to other treatment approaches.

UPPP is used in combination with AT, most commonly in children with neurological impairment. In 2002, a retrospective study of 15 patients was undertaken to determine the effectiveness of using AT and UPPP in patients with neurologic impairment and OSA. Improvement in the symptoms of OSA was found in 87% of patients, with the mean lowest oxygen saturation significantly improved from 65% preoperatively to 85% postoperatively (*P* = 0.005). However, 23% of these patients demonstrated the need for further airway intervention during follow-up. The authors concluded that AT with UPPP should be considered in patients with moderate-to-severe OSA with neurological impairment and should be limited to the posterior pharyngeal area [47]. Controlled studies of adjuvant UPPP surgery in pediatric patients with OSA, however, have not yet been undertaken.

Lingual tonsil hypertrophy (LTH) is a common anatomic cause of persistent OSA in children. The surgical procedure of lingual tonsillectomy is performed for children with persistent OSA due to LTH after AT. In 2017, the findings of a systematic review and meta-analysis of the clinical effectiveness of lingual tonsillectomy for persistent pediatric OSA identified five controlled studies, from which four studies underwent meta-analysis [48]. Lingual tonsillectomy for LTH was found to be a safe and effective adjunctive surgical treatment for persistent OSA in patients after AT. Lingual tonsillectomy was found to reduce the AHI and increase oxygen saturation, without the use of CPAP. Rate of adverse events including hemorrhage and postoperative air way obstruction was similar to A&T [48]. Because of the small number of patients studied, no recommendations can be made for the routine use of lingual tonsillectomy for LTH in persistent pediatric OSA.

Tongue-base reduction and base-of-tongue suspension are rare procedures that may be performed with hyo-epiglottoplasty for the treatment of severe OSA. In 2017, a systematic review and meta-analysis on tongue surgery for pediatric OSA identified 11 studies that included 116 children. Most of the children requiring this form of surgery had congenital syndromes with craniofacial abnormalities, such as severe micrognathia or Pierre Robin sequence, or serious comorbidities. Surgical procedures included tongue-base reduction (*N* = 114), HGN stimulation (*N* = 1), and tongue suspension (*N* = 1). The surgical procedures reduced the mean preoperative and postoperative AHI from 16.9 ± 12.2/h to 8.7 ± 10.6/h (by 48.5%) in all 114 patients. Nonsyndromic children and children with a BMI < 25 kg/m^2^ showed the most improvement in AHI [49]. More than 90% of patients who underwent tongue-base reduction surgery had previously undergone AT. Tongue-base reduction carries with it the potential for damage to neurovascular structures. The number of pediatric patients studied makes it clear that this procedure should not be considered first-line therapy, nor should it be performed simultaneously with AT.

Supraglottoplasty is often used in younger children with OSA (age less than 1 or 2 years), with congenital laryngomalacia, or in cases of later-onset laryngomalacia typically found only in the sleeping state (sleep-dependent laryngomalacia). Camacho et al. performed a systematic review and meta-analysis in 2016 of 13 studies, which showed a mean decrease in AHI of 16.4 (from 20.4 to 4.0) in congenital laryngomalacia and 10.7 (from 14 to 3.3) in sleep-dependent laryngomalacia. Of note, all children with sleep-dependent laryngomalacia were diagnosed by drug-induced sedation endoscopy in the studies reviewed [50]. Lee et al. performed a similar review of 11 studies. Pooled results of all types of laryngomalacia showed a mean decrease in AHI of 8.9. Secondary analysis showed that for patients with laryngomalacia, there was no significant difference in effect if the surgery was performed as a primary intervention or secondarily after another type of airway surgery, such as AT [51]. Digoy et al. reported postoperative coughing and dysphagia [52]. These studies suggest that supraglottoplasty is an effective treatment for patients with underlying laryngomalacia, although it is still not 100% effective [51].

As in medical therapy, if a patient also has nasal allergies, additional procedures may be employed. Most commonly, this includes an inferior turbinate reduction, but could also include septoplasty or, in select cases, sinus surgery. Studies are typically small in size and often examine many variations of nasal surgery, making assessment difficult. As such, a recent review found no significant improvement in AHI; however, improvements were demonstrated on the Epworth Sleepiness Scale and Respiratory Disturbance Index [53]. Nonetheless, nasal surgeries may play a role in improving tolerance of CPAP, potentially through decreasing nasal airway resistance and decreasing the pressure of CPAP needed [54]. Much of this research does not focus on pediatric patients, and more research with greater sample sizes are needed before this can be recommended widely as treatment.

Maxillomandibular advancement (MMA) is performed by an oro-maxillo-facial surgeon that uses Le Fort I maxillary and sagittal split mandibular osteotomies to enlarge the skeletal structure of the pharyngeal airspace. Surgical success in these cases is typically defined as a reduction in the AHI > 50% from baseline and below 20 in total. Numerous meta-analyses have shown effectiveness in reducing AHI in adult patients, with surgical success rates typically reported around 75% or higher when assessed in the postsurgical year [55]. Boyd et al. performed investigated quality of life measurements, including the Epworth Sleepiness Scale, Functional Outcomes of Sleep Questionnaire, and Medical Outcomes Study 36-Item Short Form Health Survey, all of which showed statistically significant improvement 4 to 12 months after MMA, even in the presence of prior CPAP usage [56]. Short-term data are very promising, but emerging data on longer-term follow-up greater than 8 years does show a trend towards at least a ten-point increase in AHI after the immediate improvement. In the case of one cohort by Vigneron et al., the AHI increased to 25.5 at approximately 13 years after MMA, which would qualify as surgical failure by typical definitions [57, 58]. In a cohort of 9 patients from an initial cohort of 12 followed for a median of 19 years, successfully treated patients showed no significant symptoms of sleepiness or change in quality of life measures covering headaches, concentration, insomnia, or snoring despite two patients relapsing to prior to procedure AHI values [59].

Data in pediatric patients are limited with this surgical approach. A patient with significant malocclusion, retrognathia, a large overjet, and teeth crowding underwent MMA at 11 years of age. She maintained her facial profile, occlusion, and experienced a 68% reduction in AHI even up to 4 years after surgery with no increase in sleepiness [60]. It has been reported for children with identifiable syndromes associated with micrognathia and mandibular ankylosis. Many research efforts have focused on finding the optimal cephalometric analysis and values to track both in a presurgical and postsurgical fashion, and measurements used with success have included total pharyngeal airway volume, minimal axial area, and the mandibular occlusive plane [61, 62]. This suggests that correctible dental defects are an important part of evaluation before referring for MMA. The timing of MMA is complicated in children, as the optimal age to perform such an intervention is unknown, although late adolescence has been suggested by some experts [63]. To date, no randomized trials or major systematic reviews have been performed for MMA in the pediatric population, which limits the ability to extrapolate information.

Tracheostomy is usually reserved for the treatment of severe OSA that does not respond to any other form of treatment; however, children with craniofacial disorders, including severe microglossia or micrognathia, and severe morbid obesity who do not have adenotonsillar hypertrophy may require tracheostomy as the primary treatment for OSA. A recent systematic review of the literature for controlled studies on the outcome of tracheostomy for the treatment of pediatric OSA identified 11 studies in which 196 pediatric patients underwent tracheostomy (mean age, 4.2 years; age range, newborn to 18 years). Following tracheostomy, the AHI index showed a 97% reduction in two studies, and all patients showed significant improvement in breathing. The authors of this study concluded that based on studies with preoperative and postoperative data, tracheostomy was a successful treatment for OSA in this pediatric patient population [64]. However, no conclusions can be drawn from this study due to the small number of patients included and the lack of data included in the published studies.

## 6. High-Flow Nasal Cannula Therapy

In the past decade, HFNC treatment became increasingly used as noninvasive approach to respiratory support for acute and chronic respiratory failure in patients of all age, including children and neonates [65]. HFNC therapy has been used in neonates and children with a range of respiratory disease and has been shown to be as effective as nasal CPAP for the treatment of respiratory distress syndrome in neonates [66].

The advantage of HFNC therapy is that very high flows can be delivered as the cannula heats and humidifies the oxygen and air mixture. The mechanisms involved in HFNC therapy include oxygenation of previous nonventilated areas in the upper airways with an inspiratory flow rate that is greater than normal, which reduces upper airway collapse and results in a continuous positive pressure in the airways [65].

In 2015, a study included five cases of OSA in children without adenotonsillar hypertrophy who were treated with HFNC therapy. HFNC therapy was well tolerated and resulted in significant improvement in the AHI and oxygen saturation levels [65]. An advantage of HFNC therapy was that it could be used in the home. Large-scale randomized controlled trials are required to determine whether HFNC may be used as an alternative to CPAP for the management of OSA in children.

## 7. Hypoglossal Nerve Stimulator Therapy

The hypoglossal nerve (HGN) can be stimulated using an implantable device that can sense respiratory patterns. During inspiration, the HGN stimulator delivers electrical impulses to the medial segment of the hypoglossal nerve during inspiration, resulting in protrusion of the tongue [67]. Upper airway stimulation with the HGN stimulator has recently been shown to be effective in the management of adults with moderate-to-severe OSA [68]. The HGN stimulator has also been shown to be effective in pediatric patients with Down's syndrome and OSA. The use of the HGN stimulator requires surgical placement via a single medial chest incision [67].

A recently reported case series included patients with Down's syndrome who underwent HGN stimulator implantation. The study included six children and adolescents (12 to 18 years of age) who had severe OSA with an AHI > 10 events/hour, despite previous AT. In all six patients, HGN stimulator therapy was well tolerated with mean use of 5.6–10.0 hours per night, resulting in significant improvement in the symptoms of OSA. At follow-up between 6 and 12 months, the patients had a 56% and 85% reduction in AHI and improved quality of life, respectively [69]. HGN stimulation is a potential therapeutic option for children with Down's syndrome who have refractory OSA following AT and who are unable to tolerate the use of CPAP devices. Applicability of these findings is limited due to the lack of long-term data, as well as the battery life of individual units.

## 8. Adjuvant Therapy: Positional Therapy

In approximately 56% to 75% of adult patients with OSA, the duration and frequency of episodes of apnea are affected by body position, referred to as position-dependent or positional OSA [70]. Some pediatric studies have found similar effects of sleeping position on OSA severity, with more frequent events when sleeping in the supine position [71, 72]. Devices to avoid supine sleep include shirts, bands, backpacks filled with different sized balls (from tennis balls to footballs) swimming aids, or long plastic pipes to the more sophisticated chest vests, chest straps, neck braces, and “smart” electronic devices that vibrate when the wearer is in certain positions [70, 73, 74]. Commercially available pillows and belts can be combined with elevating the head of the bed [74, 75]. Positional therapy has not been well studied in children, and studies in adults have been small and nonrandomized.

The findings from a recently reported prospective study on treatment response and compliance in positional OSA in 28 patients with the use of a sleep-positioning pillow evaluated fatigue, sleepiness, and quality of sleep quality using the Epworth Sleepiness Scale (ESS), the Pittsburgh Sleep Quality Index (PSQI), the fatigue severity scale (FSS), and the Function Outcomes of Sleep Questionnaire (FOSQ) from the beginning of the study and at one month and six months. The PSG findings showed significant effects after one night, which were sustained for one month according to the indices of PSQI (*P* < 0 .001), ESS (*P* < 0.005), and FOSQ (*P* < 0.001). Also, patient compliance and overall satisfaction were high at the one-month and six-month follow-ups [76]. This study did not evaluate pediatric patients.

A recently reported study evaluated a 1-month trial period with a sleep position trainer (SPT) in patients with positional OSA. In the 79 adult patients who completed the study protocol, adherence was found to be 95 ± 8%, and 50% of patients were responders with a reduction on the respiratory event index [77]. This study did not evaluate pediatric patients.

A systematic review and meta-analysis evaluated the efficacy of several new generation devices for positional therapy (devices producing vibrating stimuli that prevent changing to the supine position) for patients with positional OSA. Combined data from four randomized controlled trials showed a 54% reduction in the AHI and an 84% reduction in total sleeping time in the supine position [70].

Another meta-analysis and systematic review found that while CPAP was more effective than positional therapy in improving the severity of OSA in adults, adherence to the electronic positional therapy devices was higher than that for CPAP in the short term [78].

Studies to date have not examined the effect of positional therapy on children with OSA or compared it with other modalities to treat OSA. Given its potential benefit in the adult population, positional therapy might be useful as adjunctive therapy in the management of pediatric patients with supine predominant OSA. It should be kept in mind that the etiology of pediatric OSA varies widely depending on the age group. This issue should be an important focus of future research.

## 9. Conclusions

**Figure 3:**
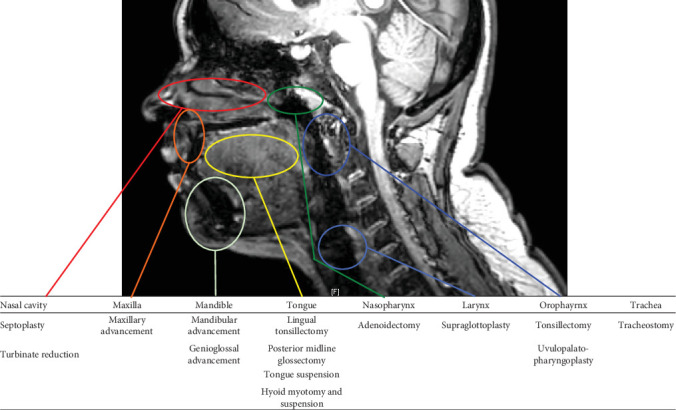
Sites of obstruction and potential surgical approaches for remedy.

**Figure 4:**
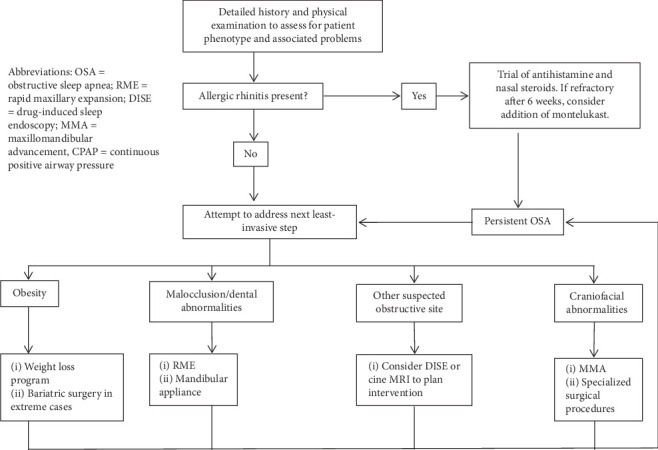
Suggested treatment algorithm persistent OSA after adenotonsillectomy or patients intolerant to CPAP.

Evidence-based guidelines support the use of CPAP as an effective first-line treatment of OSA in children without adenotonsillar hypertrophy; however, this is complicated by low tolerance or refusal of treatment. As such, this review illustrates several practical ideas for clinical practice: (1) weight management through exercise and dietary advice for patients who are overweight with OSA can reduce symptoms and morbidity; (2) treatment of underlying metabolic causes of weight gain (such as acromegaly and hypothyroidism); (3) surgical treatments if targeted properly, even for patients with severe OSA who are overweight; (4) identifying level of obstruction through the use of DISE and choosing appropriate adjuvant surgery or orthodontic procedures for long-lasting treatment effect; and (5) emerging treatments such as oxygen therapy through HFNC and pharmacotherapy which are still awaiting further support through clinical trials and long-term follow-up studies of adverse effects. There are several secondary non-CPAP treatments that can be initiated for pediatric obstructive sleep apnea as shown in Figures 3 and 4. Each has had varying levels of success. Direct comparison of the treatments listed in this review to CPAP is an important next step in research to determine how successful these treatments are compared with the standard of care. Our review supports the idea that treatment for pediatric sleep apnea is best when individualized for the patient and their underlying phenotype of obstructive sleep apnea.

## Figures and Tables

**Table 1:** Orthodontic devices/procedures addressing OSA in children.

Author (date)	Age	*N*	Type of intervention	Prior procedure performed	Type of study	Mean pre/post AHI
Al-Jewair, Gaffar, and Flores-Mir (2016)	<18 yrs	542	Varied orthodontic appliances	Not reported	Systematic review	Not reported
Camacho et al. (2016)	<18 yrs (mean 7.6)	314	RME	Some groups with prior adenotonsillectomy	Systematic review and meta-analysis	8.9/2.7 (70% reduction)
Huynh, Desplats, and Almeida (2016)	<18 yrs	238 (only 39 in OMA group)	OMA, RME, or MA and RME	None (excluded)	Systematic review and meta-analysis	13.1% in OMA, unable to calculate for RME and MA with RME
Nazarali et al. (2015)	<16 yrs	134	MAD	None (excluded)	Systematic review and meta-analysis	Not calculated due to high risk of bias

AHI: apnea/hypopnea index; MAD: mandibular advancement device; RME: rapid maxillary expander; OMA: orthopaedic mandibular advancement; MA: myofunctional appliances.

**Table 2:** Surgical procedures addressing OSA in children other than tonsilloadenoidectomy.

Author (date)	Age (mean age)	*N*	Type of procedure	Prior procedure performed	Type of study	Mean pre/post AHI
Rivero and Durr (2017)	<18 years (9.5)	132	Lingual tonsillectomy	T+A	Systematic review and meta-analysis	12.291/5.653 (54% reduction)
Camacho et al. (2017)	<18 years (10.8)	116	Base of tongue reduction (114), tongue suspension (1), and HNS (1)	>90% T+A	Systematic review and meta-analysis	16.9/8.7 for tongue-base reduction (48.5% reduction)
Fray et al. (2018)	<18 years (11.1)	196	Tracheostomy	Not reported	Systematic review and meta-analysis	34.2/0.75 (98% reduction)
Camacho et al. (2016)	1 month to 12.6 years (mean not reported)	138	Supraglottoplasty	Excluded	Systematic review and meta-analysis	20.4/4 (80% reduction) in congenital laryngomalacia 14/3.3 (76% reduction) in sleep-exclusive larnygomalacia
Lee et al. (2016)	2.4 months to 7.4 years (3.7)	121	Supraglottoplasty	AT or lingual tonsillectomy in some patients	Meta-analysis	8.9 mean decrease (mean pre/post not included)
Noller et al. (2018)	<18 years	376	MAD or mandibular advancement surgery	None (excluded)	Systematic review and meta-analysis	41.1/4.5 (89.1% reduction)

T+A: tonsillectomy and adenoidectomy; AT: adenotonsillectomy; MAD: mandibular advancement device.
